# Traumatic Serratus Anterior Muscle Tear Presenting as Isolated Posterior Shoulder Pain in a Professional Volleyball Athlete: A Case Report

**DOI:** 10.7759/cureus.115372

**Published:** 2026-08-28

**Authors:** Inês Camarinha, Diogo Rodrigues, Catarina Afonso, Rui Domingues

**Affiliations:** 1 Physical Medicine and Rehabilitation, Unidade Local de Saúde São José, Lisbon, PRT; 2 Orthopedics and Traumatology, Hospital Ortopédico de Sant'Ana, Lisbon, PRT

**Keywords:** muscle tear, serratus anterior muscle injury, shoulder pain, sports-related injury, volleyball injury

## Abstract

Serratus anterior muscle injuries are rare, most commonly reported in rowing and overhead throwing athletes. These injuries typically present with anterolateral chest wall pain and a palpable mass. Atypical presentations may lead to diagnostic challenges, particularly when symptoms mimic shoulder pathology. We report the case of a 26-year-old male professional volleyball player who presented with acute-onset posterior shoulder pain following a serving motion. The pain persisted for two weeks and was exacerbated by overhead movements, particularly during spiking. Physical examination revealed localized posterior shoulder pain with mild limitation of external rotation, while rotator cuff testing was normal. Initial clinical suspicion included posterosuperior labral injury or posterior internal impingement. Point-of-care ultrasonography of the shoulder showed no significant abnormalities, and magnetic resonance imaging (MRI) was subsequently requested, which revealed a traumatic partial-thickness tear of the serratus anterior muscle, with a fluid-filled gap measuring approximately 13 mm and associated edema extending posteriorly beneath the rhomboid musculature. No intra-articular or rotator cuff pathology was identified. This case illustrates a rare serratus anterior injury in a volleyball athlete, a mechanism seldom reported in the literature. Unlike typical presentations, the patient had isolated posterior shoulder pain resulting from posterior tracking of edema, mimicking intra-articular pathology and representing a potential diagnostic pitfall. MRI was key to establishing the diagnosis and excluding other causes, and the patient was successfully managed with a conservative, structured rehabilitation protocol. Serratus anterior muscle tear should be considered in the differential diagnosis of posterior shoulder pain in overhead athletes, particularly when clinical findings do not support intra-articular pathology. Early recognition and appropriate imaging are essential to avoid misdiagnosis and ensure optimal management.

## Introduction

Injuries of the serratus anterior muscle are rare in sports medicine, with fewer than 10 cases reported in the literature to date, most involving rowing, golf, baseball, and other overhead throwing athletes [1-5]. Repetitive scapulothoracic loading, forceful trunk rotation, and eccentric muscular contraction are thought to generate substantial mechanical stress on the muscle and its costal attachments, predisposing athletes to injury [1-5].

The serratus anterior plays a key role in scapular stabilization, protraction, and upward rotation, contributing to efficient kinetic chain transfer during overhead athletic activities [6]. In volleyball, serving and spiking require explosive trunk rotation combined with rapid scapular protraction and shoulder internal rotation, generating biomechanical demands similar to those observed in throwing sports. These repetitive high-velocity movements may place considerable stress on the serratus anterior, making it susceptible to acute overload injury [4,5].

Reported serratus anterior injuries typically present with anterolateral chest wall pain, swelling, and occasionally a palpable mass [1,2]. Although traumatic rupture or avulsion of the serratus anterior has been sporadically reported, atypical clinical presentations remain poorly characterized, and symptom distribution may vary according to the extent of injury and the pattern of fluid and edema propagation within adjacent soft tissues. Isolated posterior shoulder pain closely mimicking intra-articular pathology has rarely been emphasized in the literature.

To date, no report has described a serratus anterior injury occurring in a volleyball player, and none has characterized a presentation limited to isolated posterior shoulder pain in the absence of chest wall symptoms. Because this pattern can closely mimic intra-articular pathology, failure to recognize it may lead to unnecessary or misdirected investigation of the glenohumeral joint. We therefore report this case to document this atypical clinical presentation, characterize its MRI features, and highlight serratus anterior injury as a diagnostic consideration in overhead athletes with unexplained posterior shoulder pain.

## Case presentation

History and physical examination

We present a 26-year-old male professional volleyball player who presented with acute-onset pain in the posterior aspect of his dominant (right) shoulder, sustained during competitive play. The patient reported that the symptoms began during a forceful serving motion, when he experienced a sudden sharp, stabbing, "tearing" sensation localized to the posterior shoulder and medial scapular region. Despite the injury, he was initially able to continue the match; the pain persisted over the following two weeks and progressively impaired his athletic performance, prompting further evaluation.

The patient described pain that was specifically aggravated by overhead activities, particularly attacking and spiking movements requiring shoulder elevation, abduction, external rotation, and rapid internal rotation. Daily activities were relatively well tolerated, whereas high-demand sporting maneuvers consistently reproduced his symptoms. He denied anterior shoulder pain, instability, and weakness of the upper extremity.

The patient had competed at the professional level, playing as an opposite hitter (attacker), with a training load of approximately 10-15 hours per week. He had no relevant comorbidities: no history of previous shoulder, elbow, or scapulothoracic injuries, no regular medication use, no known drug allergies, and no other documented health problems. There was no reported direct traumatic impact to the shoulder, and the mechanism of injury was consistent with sudden muscular overload during an explosive overhead motion.

On physical examination, there was no local swelling or visible tumefaction over the affected right shoulder or scapular region; however, palpation elicited discomfort over the rhomboid musculature. Active and passive shoulder range of motion were largely preserved and essentially symmetric to the contralateral (left) side, with the exception of a discrete deficit of approximately 20° in external rotation on the right side. Rotator cuff strength was preserved (Medical Research Council grade 5/5). Provocative tests for rotator cuff pathology, glenohumeral instability and subacromial impingement were all negative. The combined maneuver of forced abduction and external rotation reproduced posterior shoulder pain, with a positive posterior impingement sign. The crank test and the dynamic posterior impingement test also reproduced posterior shoulder discomfort but did not demonstrate findings clearly suggestive of labral pathology, and there was no painful click or instability. Scapular assessment revealed a discrete asymmetry of scapulohumeral kinesis on the affected side during repeated forward elevation and lowering of the arm, compared with the contralateral side. No significant scapular malposition was observed at rest, and there was no evidence of scapular winging during wall push-up testing, making long thoracic nerve dysfunction unlikely. Neurological examination of the affected upper extremity was normal. Based on the presenting complaint and these examination findings, the leading differential diagnoses were posterior internal impingement and posterosuperior labral injury.

Imaging findings

Point-of-care ultrasonography performed at the initial consultation showed no significant abnormalities, and MRI of the shoulder was subsequently obtained for definitive diagnostic characterization. MRI demonstrated no osseous abnormalities, fracture, or bone marrow edema, and the rotator cuff tendons and glenohumeral joint were normal in signal and morphology, with no evidence of labral or intra-articular pathology. On fat-suppressed T2-weighted sequences, a focal partial-thickness tear was identified involving the superior and anterior portion of the serratus anterior muscle, with a fluid-filled gap measuring approximately 13 mm that demonstrated marked, fluid-like hyperintensity relative to adjacent normal muscle (Figures 1-2). Surrounding the defect, the muscle belly showed a more diffuse zone of intermediate-to-high T2 signal intensity, consistent with perilesional edema, which extended along the deep surface of the muscle and tracked posteriorly beneath the rhomboid musculature and adjacent to the paraspinal muscles. The latissimus dorsi, teres major, and trapezius muscles demonstrated normal signal intensity throughout, without evidence of injury. 

**Figure 1:**
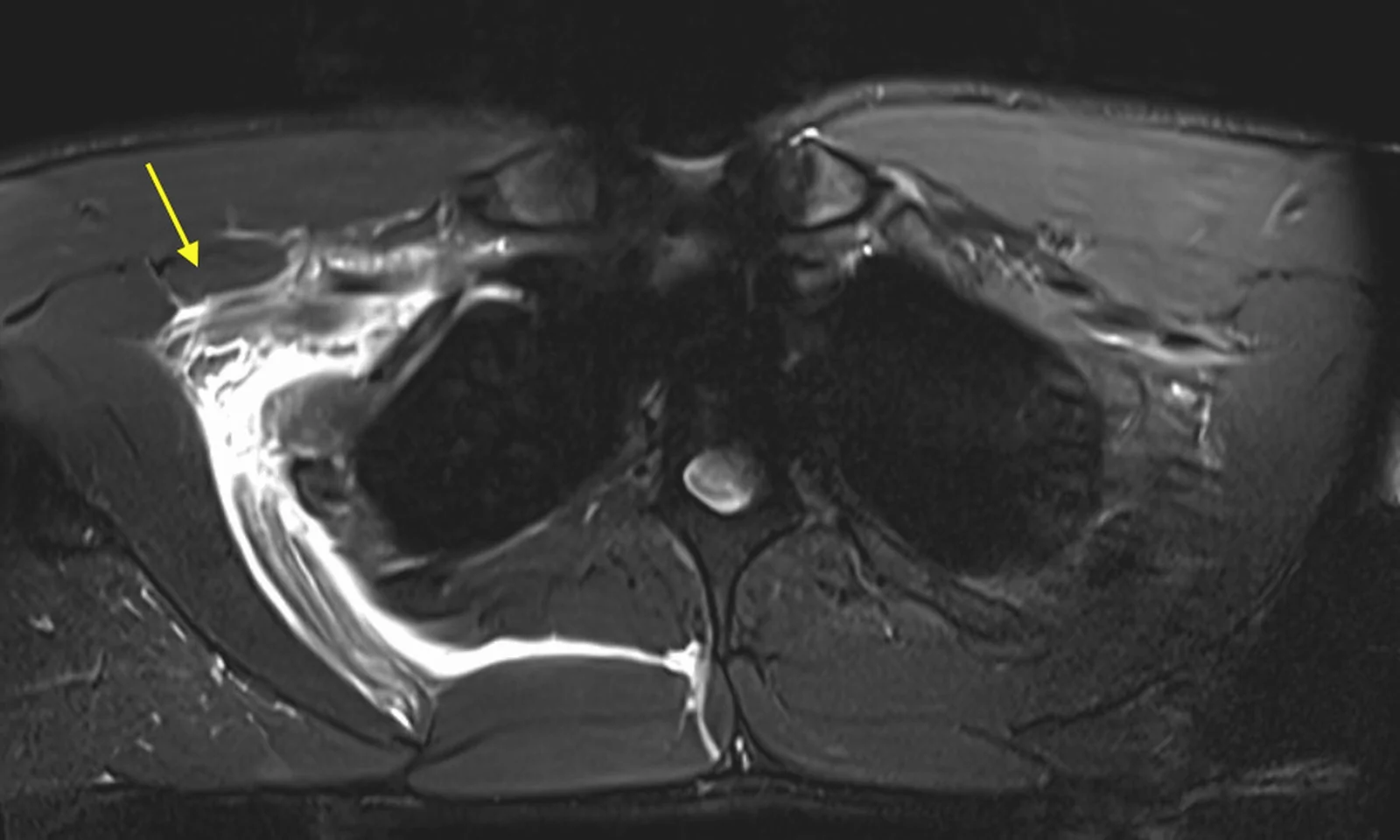
Axial MRI view Axial fat-suppressed T2-weighted MRI of the right shoulder demonstrating a partial-thickness tear of the serratus anterior muscle with an associated fluid-filled defect (arrow) and surrounding edema that tracks posteriorly beneath the rhomboid musculature.

**Figure 2:**
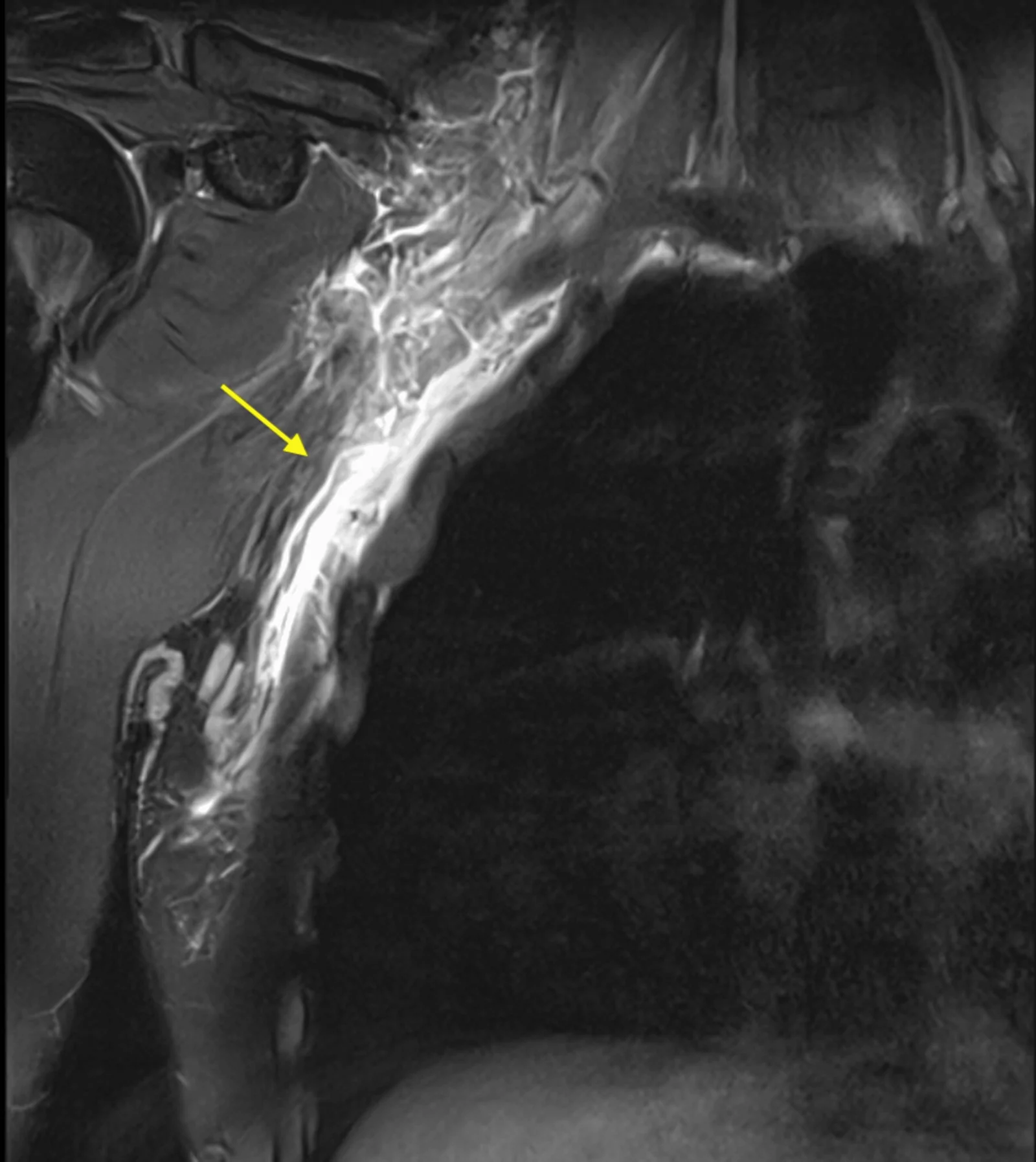
Coronal MRI view Coronal fat-suppressed T2-weighted MRI confirming partial-thickness tear (arrow) with edema extending along the serratus anterior muscle belly.

Sagittal images further demonstrated the longitudinal extent of the tear and associated edema along the costal attachments of the serratus anterior, corroborating the axial and coronal findings (Figure 3). The clinical and imaging findings together established a diagnosis of traumatic partial-thickness tear of the serratus anterior muscle with associated posterior fluid tracking. 

**Figure 3:**
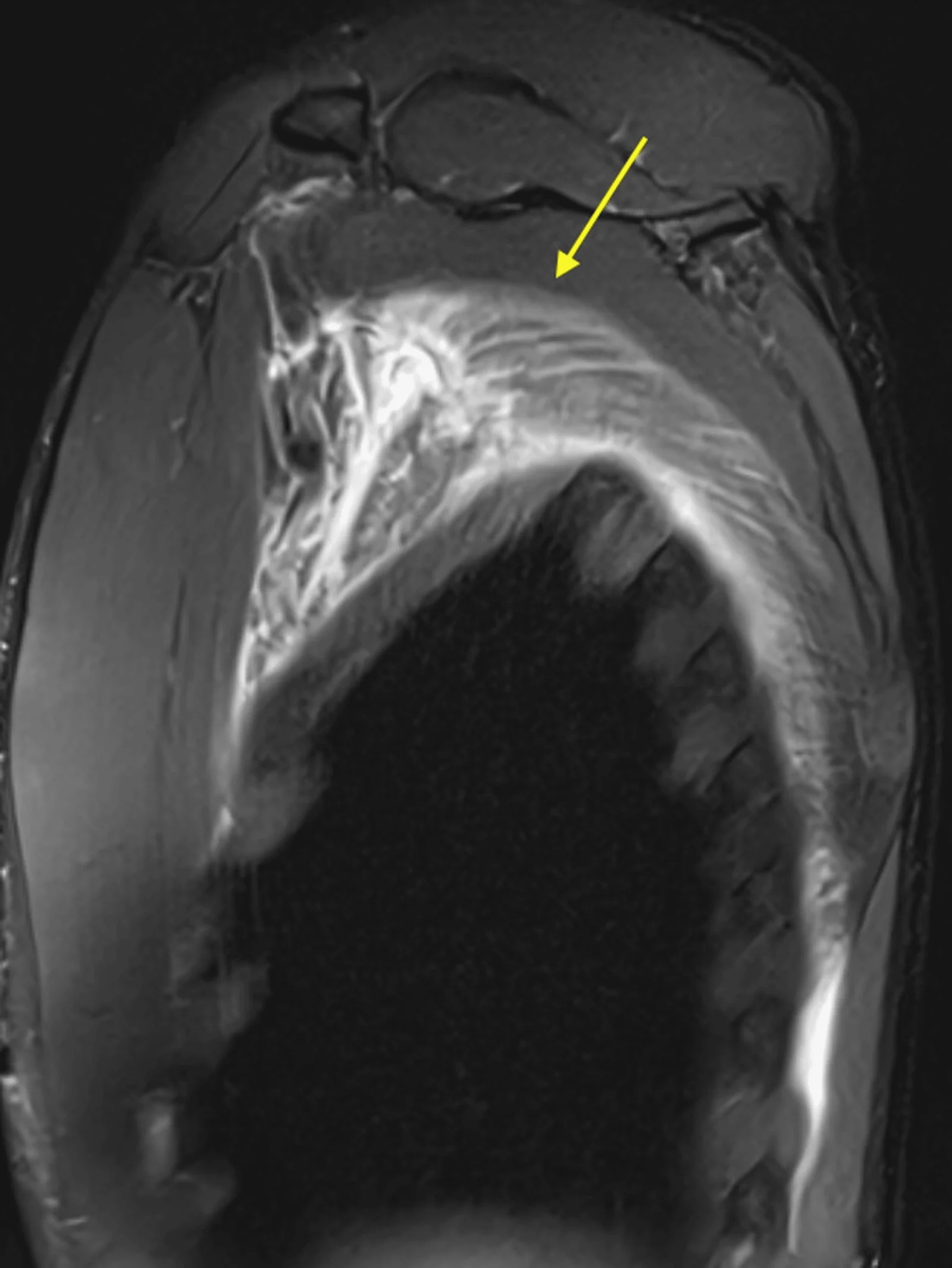
Sagittal MRI view Sagittal fat-suppressed T2-weighted MRI showing the longitudinal extent of the tear (arrow) and associated edema along the costal attachments of the serratus anterior muscle.

Treatment and outcome

The patient received anti-inflammatory and analgesic therapy for pain control in the initial days after injury, achieving partial symptom relief, and subsequently began a structured, progressive rehabilitation program directed at restoring normal scapulothoracic mechanics. Treatment initially focused on pain control, protection of the injured muscle, and maintenance of pain-free shoulder mobility through activity modification, relative rest from overhead sports activities, cryotherapy as needed, and gentle active-assisted and active range-of-motion exercises.

As symptoms improved, rehabilitation progressed to scapular stabilization exercises and strengthening of the periscapular musculature, including the serratus anterior, trapezius, and rhomboid muscles, with the specific aim of correcting the scapular dyskinesis identified on examination and re-establishing coordinated scapulothoracic motion. This phase incorporated closed-chain scapular control drills, wall slides, serratus punches, dynamic scapular protraction exercises, and progressive resistance training with elastic bands, alongside rotator cuff strengthening and kinetic chain exercises involving the trunk and lower extremities. With continued clinical improvement, the program advanced to sport-specific strengthening, plyometric exercises, and a graduated hitting and serving progression, beginning with low-intensity volleyball-specific movements and advancing to full-effort actions as tolerated.

The patient's symptoms improved progressively throughout rehabilitation. He resumed team training after three weeks and returned to competitive play after four weeks. At follow-up visits at week one, week two, one month, three months, and six months after his return to competition, he remained symptom-free, with full, pain-free shoulder range of motion and no recurrence during volleyball-specific activity.

## Discussion

Serratus anterior injuries are extremely uncommon, with most available evidence limited to isolated case reports [1-5]. The majority of cases involve rowers or throwing athletes, where repetitive high-load scapulothoracic motion plays a central role in injury development [1-5]. Repetitive loading, fatigue, or acute overload may lead to failure at the costal attachments or within the muscle fibers, as previously described [1,2]. Unlike previously reported cases, which typically present with anterolateral chest wall pain and a palpable mass [1,2], this patient presented with isolated posterior shoulder pain.

Surface electromyographic studies of the volleyball serve and spike have demonstrated intense serratus anterior activity throughout the wind-up, cocking, acceleration, and follow-through phases of the motion [7]. During the wind-up and cocking phases, as the arm is raised into abduction and external rotation, the serratus anterior contracts to upwardly rotate and protract the scapula, positioning the glenoid to support the elevating humerus and providing a stable scapulothoracic platform against which the rotator cuff and deltoid can act. During the acceleration phase, forceful trunk rotation and rapid scapular protraction combine to transfer energy from the lower extremities and trunk, through the scapulothoracic articulation, into the upper limb - a kinetic chain in which the serratus anterior is the principal muscular link. In the follow-through phase, as the arm decelerates after ball contact, the muscle reverses role and contracts eccentrically to control scapular retraction and dissipate the residual momentum of the swing. It is this repeated cycle of concentric protraction under load followed by high-velocity eccentric deceleration, occurring hundreds of times per training session in a professional athlete, that is thought to render the muscle particularly susceptible to fatigue-related microtrauma and, ultimately, acute fiber failure during a single forceful effort. Disruption of this coordinated activation pattern is also a recognized contributor to scapular dyskinesis [8], as observed on examination in our patient. We hypothesize that the serving motion in our patient generated a combination of rapid scapular protraction, trunk rotation, and eccentric muscular loading that, superimposed on the cumulative effect of a high weekly training volume, exceeded the tensile capacity of the muscle fibers and produced the observed partial-thickness tear.

Even in the limited number of cases reported in overhead athletes, symptoms were generally localized to the lateral chest wall, facilitating clinical suspicion of a scapulothoracic muscular injury [1-5]. In contrast, our patient presented exclusively with posterior shoulder pain and lacked the characteristic chest wall findings typically described in the literature, which initially suggested more common shoulder disorders encountered in volleyball players, including posterosuperior labral injury and posterior internal impingement. MRI demonstrated that this atypical distribution was explained by posterior tracking of edema from a substantial partial-thickness tear beneath the rhomboid and paraspinal musculature - a distinct clinicoradiological pattern not previously emphasized in this condition and a potential source of diagnostic error when evaluating overhead athletes with suspected labral pathology or posterior internal impingement.

This posterior pattern of fluid propagation can be explained anatomically. The serratus anterior is structurally and functionally divided into superior, middle, and inferior parts, with the superior slips converging on the superior angle of the scapula in close proximity to the insertion of the levator scapulae and the origin of the rhomboid minor [6], and its investing fascia is contiguous with the deep fascial planes of the periscapular musculature. A tear confined to this superior portion, as identified on MRI in our patient, would therefore be expected to communicate preferentially with the posterior scapulothoracic fascial compartment. This contrasts with injuries of the mid or inferior muscle belly - the site more commonly affected in previously reported cases [1-5] - which lies closer to the lateral chest wall along the lower ribs and is anatomically predisposed to produce the anterolateral swelling and palpable mass classically described in this condition. This anatomical distinction offers a plausible mechanistic explanation for why our patient's presentation diverged so markedly from prior reports.

Reported management strategies and outcomes vary considerably across the published sports-related cases, reflecting differences in injury severity. Gaffney's rower recovered after approximately four weeks of activity restriction and physiotherapy [3], and Carr et al.'s collegiate rower achieved full recovery after a two-month graduated physical therapy program [2]. Winther and Øhlenschlæger's golfer, diagnosed by ultrasonography, also recovered fully after a scapula-stabilizing rehabilitation program [1]. In contrast, Otoshi et al.'s adolescent pitcher, who sustained a complete avulsion of the serratus anterior from its costal insertion, required surgical repair and did not return to competition until seven months postoperatively [4], while Futamura et al.'s high-school pitcher, whose injury was limited to the lower muscle fibers on ultrasonography, returned to competitive throwing after 32 days of conservative management with local compression and progressive rehabilitation, without recurrence over six months of follow-up [5]. This spectrum suggests that recovery time tracks more closely with the anatomical extent of muscle disruption - particularly whether the injury represents a partial tear or a complete avulsion requiring surgical fixation - than with any single diagnostic or sporting factor. Consistent with this pattern, our patient's partial-thickness tear, without evidence of complete costal avulsion or muscle retraction, was successfully managed nonoperatively, with return to competitive play at four weeks.

Beyond the sports medicine literature, a small number of older reports have described traumatic serratus anterior rupture or avulsion in non-athletic settings, including direct trauma and acute overload during heavy lifting [9-12]. These cases typically presented with scapular winging rather than pain as the dominant feature, and most were managed conservatively with satisfactory functional outcomes. These cases differ mechanistically from the overload injury described here, but confirm that structural failure of the serratus anterior - separate from long thoracic nerve palsy - is a rare, recognized entity beyond sports medicine as well. Sport-related cases remain exceptionally rare, with fewer than 10 reported to date.

MRI remains the gold standard for diagnosis, allowing detailed assessment of muscle integrity, edema, and associated soft tissue involvement [13]. Comparable imaging features - focal fiber discontinuity with a fluid-filled gap and surrounding edema on fat-suppressed T2-weighted sequences - have been illustrated in general reviews of athletic thoracic cage injury [14], reinforcing the reliability of this appearance across cases. In the present case, MRI was essential for identifying the lesion, characterizing the extent of muscle disruption, and excluding rotator cuff, labral, and other intra-articular abnormalities. Ultrasonography has also been described as a useful tool for diagnosis and serial follow-up, particularly for monitoring hematoma resolution and guiding rehabilitation progression [5], although it is operator-dependent and may be less comprehensive than MRI for evaluating the full extent of injury and edema propagation.

Most cases of serratus anterior injury are managed conservatively, with rest, progressive rehabilitation, and gradual return to sport [2,5]. Surgical intervention is rarely required but may be indicated in cases of complete avulsion with significant displacement [4]. In the present case, the patient was treated nonoperatively with a structured rehabilitation program focused on pain control, restoration of shoulder mobility, scapular stabilization, and progressive strengthening of the periscapular musculature, achieving complete resolution of pain and restoration of full shoulder function.

This report has several limitations inherent to single-case observations. Findings from one patient cannot establish the true incidence of this presentation pattern or be generalized to all volleyball players with posterior shoulder pain. No pre-injury baseline imaging or contralateral comparison sequences were available, and the diagnosis relied on clinical and MRI correlation without histological confirmation. Follow-up was limited to six months after return to competition; longer surveillance would be needed to confirm the durability of recovery and to exclude delayed recurrence or chronic scapular dysfunction.

## Conclusions

Serratus anterior tear is a rare but important cause of shoulder pain in overhead athletes and should be considered when clinical findings do not support intra-articular pathology. In this volleyball player, posterior tracking of edema from a partial-thickness tear produced isolated posterior shoulder pain that closely mimicked labral or internal impingement, a presentation not previously described for this injury. MRI was essential to confirm the diagnosis and exclude rotator cuff and intra-articular disease. Managed conservatively, the patient returned to full competition within four weeks and remained symptom-free through six months of follow-up, underscoring that even substantial partial tears of the serratus anterior can have an excellent, rapid functional outcome without surgery.
